# Drought stress modulates indirect defense via bottom‐up effects in tomato and wheat

**DOI:** 10.1002/ps.70900

**Published:** 2026-05-11

**Authors:** Mariangela Milordo, Eya Ben Hmad, Maria Flavia Pitruzzello, Carmelo Cavallaro, Giuseppe Massimino Cocuzza, Lucia Zappalà, Peng Han, Antonio Biondi, Michele Ricupero

**Affiliations:** ^1^ Department of Agriculture, Food and Environment University of Catania Catania Italy; ^2^ Yunnan FVF‐IPM International Joint Lab, School of Ecology and Environmental Science Yunnan University Kunming China

**Keywords:** drought stress, global warming, induced plant defense, *Solanum lycopersicum*, *Triticum aestivum*

## Abstract

**BACKGROUND:**

Biotic and abiotic factors induce bottom‐up effects that can be used to enhance indirect plant defenses in agroecosystems. However, the feasibility of integrating biological control with drought‐induced plant defense remains largely unexplored. We tested under laboratory conditions how three water regimes (optimal, moderate and severe drought stress) affect plant traits and the attraction of natural enemies, in infested and uninfested tomato and wheat plants. Plant morphological parameters and expression of defense‐related genes were measured. The olfactory responses of *Encarsia formosa* and *Cryptolaemus montrouzieri* to tomato volatiles, and *Aphidius colemani* to wheat volatiles were investigated.

**RESULTS:**

Moderate to severe water regimes significantly reduced stem diameter on both systems and overregulated *ASR1* and *PIN2* on tomato. *PR1* was overexpressed only under severe stress on wheat. Multiple olfactory responses among the tested natural enemies were observed. Tomato plants under moderate drought stress significantly attracted *E. formosa* without infestation. *C. montrouzieri* significantly preferred moderately water‐stressed plants when no pest occurred, but the predators chose optimally watered plants over highly drought‐stressed plants under pest infestation. *A. colemani* showed significant preference towards optimally watered plants without the host presence, except when compared to infested moderately drought‐stressed plants.

**CONCLUSIONS:**

Our results suggest that water stress and pest infestation activate plant defense mechanisms with multiple olfactory consequences on the associated beneficial arthropods. These findings could be used for implementing biocontrol strategies within the IPM context under a changing climate scenario. © 2026 The Author(s). *Pest Management Science* published by John Wiley & Sons Ltd on behalf of Society of Chemical Industry.

## Graphical abstract

Different water regimes can have a significant impact on plant morphology and defense pathways in tomato and wheat, triggering multiple olfactory choices in biocontrol agents of key insect pests. These findings could be used to implement biocontrol strategies within the IPM context under a changing climate scenario.

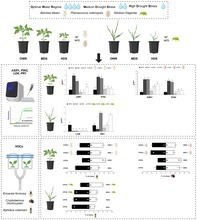

## 1 INTRODUCTION

In agroecosystems, bottom‐up effects play a role in multitrophic interactions among plants, pests and their natural enemies, where an upper trophic level is hierarchically influenced by a lower one.^1^ Plants as the primary source in ecosystems are crucial in mediating these dynamics within plant–pest–natural enemy networks.^2^ Both abiotic and biotic changes can thus trigger bottom‐up effects which affect the fitness of living organisms such as plant productivity, herbivore performances and, as a consequence, the population level of insect natural enemies. Therefore, the overall performance of ecosystem services is changed.^1^, ^3^, ^4^, ^5^ Within this context, understanding the bottom‐up effects on beneficial arthropods can help in implementing biological control programs of insect pests.^6^ Global warming is considered one of the major causes of environmental changes such as water depletion and temperature increase, which impact the incidence and distribution of agricultural pests.^4^, ^7^ Plants exposed to extreme environmental conditions undergo morphological, physiological and metabolic adaptations.^8^, ^9^ Drought generally alters a plant's nutritional profile, thereby influencing herbivore feeding rates, nutrient absorption and growth rates, with further implications for biological control agents.^4^, ^10^, ^11^ In response to water stress alone or in combination with herbivores, plants engage in a series of physiological reactions that involve significant variations in their defense metabolism.^8^, ^12^, ^13^

Tomato (*Solanum lycopersicum* L.) and wheat (*Triticum aestivum* L.) are among the most economically relevant cultivated crops worldwide, as they play a crucial role as human food commodities. However, their production is severely threatened by environmental changes and a large number of pests which are largely controlled by synthetic pesticides.^14^, ^15^ In this scenario, the use of chemical inputs (e.g. fertilizers, pesticides) must be rationalized, and more sustainable techniques such as biological control applications should be favored. Therefore, new resource optimization strategies are needed to improve the environmental sustainability of these cropping systems.^16^, ^17^ Multiple studies have highlighted that drought‐induced bottom‐up effects on tomato can have consequences on the survival and development of herbivores,^9^, ^18^ predator longevity^10^ or parasitism activity.^2^ Although plant metabolism changes are achieved by modulating several signaling pathways, such as jasmonic acid (JA), salicylic acid (SA) and abscisic acid (ABA),^19^ the effect on plant defense signaling pathways when plants face herbivore infestation in combination with drought stress has been investigated only scarcely.^13^ Even though a number of studies have revealed the impact of drought stress on plant volatiles, the behavioral response of natural enemies to plant‐emitted volatiles under drought stress with the co‐occurrence of pest infestation was addressed only on tomato.^20^ To the best of our knowledge, no previous research evaluated this aspect in wheat system.

In this framework, we hypothesized that altered water inputs and the presence of herbivore insect pests could trigger bottom‐up effects, leading to plant morphological and transcriptomic changes. For this, we tested in the laboratory the impact of three irrigation conditions (optimal water regime, moderate drought stress and high drought stress) on tomato and wheat plants infested or not. Tomato plants were infested with *Bemisia tabaci* (Gennadius) (Hemiptera: Aleyrodidae) or *Phenacoccus solenopsis* Tinsley (Hemiptera: Pseudococcidae), whereas *Sitobion fragariae* (Walker) (Hemiptera: Aphididae) was used for wheat infestation. Plant morphological parameters [height, stem diameter, root length, fresh (FW) and dry weight (DW)] were measured. The olfactory responses of *Encarsia formosa* (Gahan) (Hymenoptera: Aphelinidae) and *Cryptolaemus montrouzieri* (Mulsant) (Coleoptera: Coccinellidae) on tomato, and *Aphidius colemani* (Viereck) (Hymenoptera: Braconidae) on wheat to plant‐emitted volatiles were thus investigated. The expression levels of genes associated with major defense pathways in tomato and wheat also were assessed. The present work explores the effects of water stress and herbivore pests on their natural enemies and provides new insights for improving our understanding in biological control applications within the integrated pest management (IPM) context for tomato and wheat systems.

## 2 MATERIALS AND METHODS

### 2.1 Biological materials

#### 2.1.1 Plants

Tomato (*Solanum lycopersicum* cv. ‘San Marzano nano’) and wheat (*Triticum aestivum* cv. ‘Furio Camillo’) plants were grown from seeds in black plastic pots (diameter 7 cm, height 7 cm) filled with 25 g soil (Professional mix, Vigorplant Srl, Fombio, Italy) and 5 g of vermiculite. A single seed was placed in each pot and irrigated with 60 mL distilled water. Plant seedlings were watered twice a week (Tuesday and Friday) and fertilized with 2.5% of Multifeed® solution (Biolchim, Medicina, Italy). Plants were kept in a climatic chamber (1.54 × 3.16 × 2.43 m; Hitec, Mecter, Italy) under the following environmental conditions: 25 ± 1 °C, 60 ± 10% relative humidity (RH) and a photoperiod of 18 h:6 h, light:dark. For tomato, three water treatments were specifically designed based on the soil water retention: (i) optimal water regime (OWR), as control treatment, where plants were maintained in well‐watered conditions and initially irrigated twice a week with 20 mL water per irrigation; (ii) moderate drought stress (MDS) starting to irrigate with 10 mL water; and (iii) high drought stress (HDS) beginning to irrigate with 5 mL water. To meet water requirements associated with plant growth while maintaining drought water regimes, each week the irrigation volumes were proportionally increased as follows: +8 mL for OWR, +4 mL for MDS and +2 mL for HDS. After a 5‐week period of irrigation, the final water volumes were 60 mL for OWR, 30 mL for MDS and 15 mL for HDS. For wheat plants, water volumes were not differentiated over the 4 weeks, but instead remained standardized at 40, 25 and 20 mL for OWR, MDS and HDS, respectively. Water regimes were set for tomato and wheat plants through preliminary laboratory trials starting from a similar methodology described by Ahmed *et al*.^21^

#### 2.1.2 Herbivores

*Bemisia tabaci* or *P. solenopsis* and *S. fragariae* were chosen as herbivores for infesting tomato and wheat plants, respectively. Laboratory colonies of *B. tabaci* and *P. solenopsis* were established from individuals collected in protected tomato crops in Marina di Ragusa, Sicily (Italy) in 2022. The colonies were then reared for multiple generations on 4‐week‐old potted tomato plants in separated 50 × 50 × 50 cm ventilated plastic cages (BugDorm®, Taichung, Taiwan). Each tomato plant at the phenological growth stage of the third true leaf unfolded (BBCH 103) and grown under one of the three water regimes (OWR, MDS and HDS), was infested with 60 adult *B. tabaci* females (48‐h old) for 120 h in aerated plastic cylinders. For *P. solenopsis*, each tomato plant in each water regime was infested with 10 3^rd^‐instar nymphs for 72 h. A colony of *S. fragariae* was established from a single‐winged adult female collected in Assoro, Enna (Italy) on *T. aestivum* plants at the phenological growth stage at the third true leaf unfolded stage (BBCH 13).^22^ The colony was maintained in separated ventilated plastic cages. Wheat plants grown under the three water regimes, were infested with 20 *S. fragariae* adult females (48‐h old) for 72 h. The females were isolated from the main rearing colony. The infestation densities, insect age and exposure periods were selected based on preliminary experiments and established protocols in plant–hemipteran interaction studies^21^ to ensure consistent infestation levels while avoiding excessive plant damage. Before the beginning of each trial, all herbivores were mechanically removed from the infested plants with a soft paintbrush. Plants were then carefully inspected to ensure complete insect removal and to prevent interference between herbivore‐derived volatiles and plant defense‐induced volatiles during the assays. For each tested water regime, both with and without herbivore infestation, a total of three plants were used for molecular analysis (see Section 2.2.2). For each water regime combination, both with and without herbivore infestation, a total of four plants were used for olfactometer bioassays (see Section 2.2.3). All of the herbivore species were kept in a climatic chamber under the following environmental conditions: 25 ± 1 °C, 60 ± 10% RH and a photoperiod of 14 h:10 h, light:dark.

#### 2.1.3 Natural enemies

The olfactory choice of three natural enemies (*E. formosa*, *C. montrouzieri* and *A. colemani*) was evaluated to the volatile compounds emitted by *S. lycopersicum* and *T. aestivum* plants under different water regimes and/or herbivore infestations. *E. formosa* and *A. colemani* individuals were purchased weekly from Koppert Biological Systems (Berkel en Rodenrijs, The Netherlands). *E. formosa* pupae were provided in EN‐STRIP® packages, each containing ≈3000 parasitized whitefly pupae glued on cardboard strips. The pupae were isolated in ventilated Petri dishes (9 cm diameter). Small droplets of honey‐water solution were provided to the parasitoids on the lid of Petri dishes as a carbohydrate food source. Pupae of *A. colemani* were purchased as APHIPAR® packages containing ≈500 parasitized aphid mummies of the same age. The pupae were kept at laboratory temperature until emergence. *C. montrouzieri* specimens were obtained as pupae from the biofactory ‘Biofabbrica Insetti Utili – Ente Sviluppo Agricolo, Regione Siciliana’ (Ramacca, Italy) where insects were reared on potato sprouts infested by the citrus mealybug *Planococcus citri* (Risso) (Hemiptera: Pseudococcidae).^23^ Newly emerged 24‐h‐old *C. montrouzieri* adults were fed with jellified honey.^24^ Female individuals from all species were separated from males based on morphological features under a stereomicroscope (M205FA; Leica, Wetzlar, Germany) and isolated in ventilated plastic tubes without any host/prey for 24 h. All of the natural enemies were kept in the same environmental laboratory conditions: 25 ± 1 °C, 60 ± 10% RH and a photoperiod of 14 h:10 h, light:dark.

### 2.2 Experimental setup

**Figure 1:**
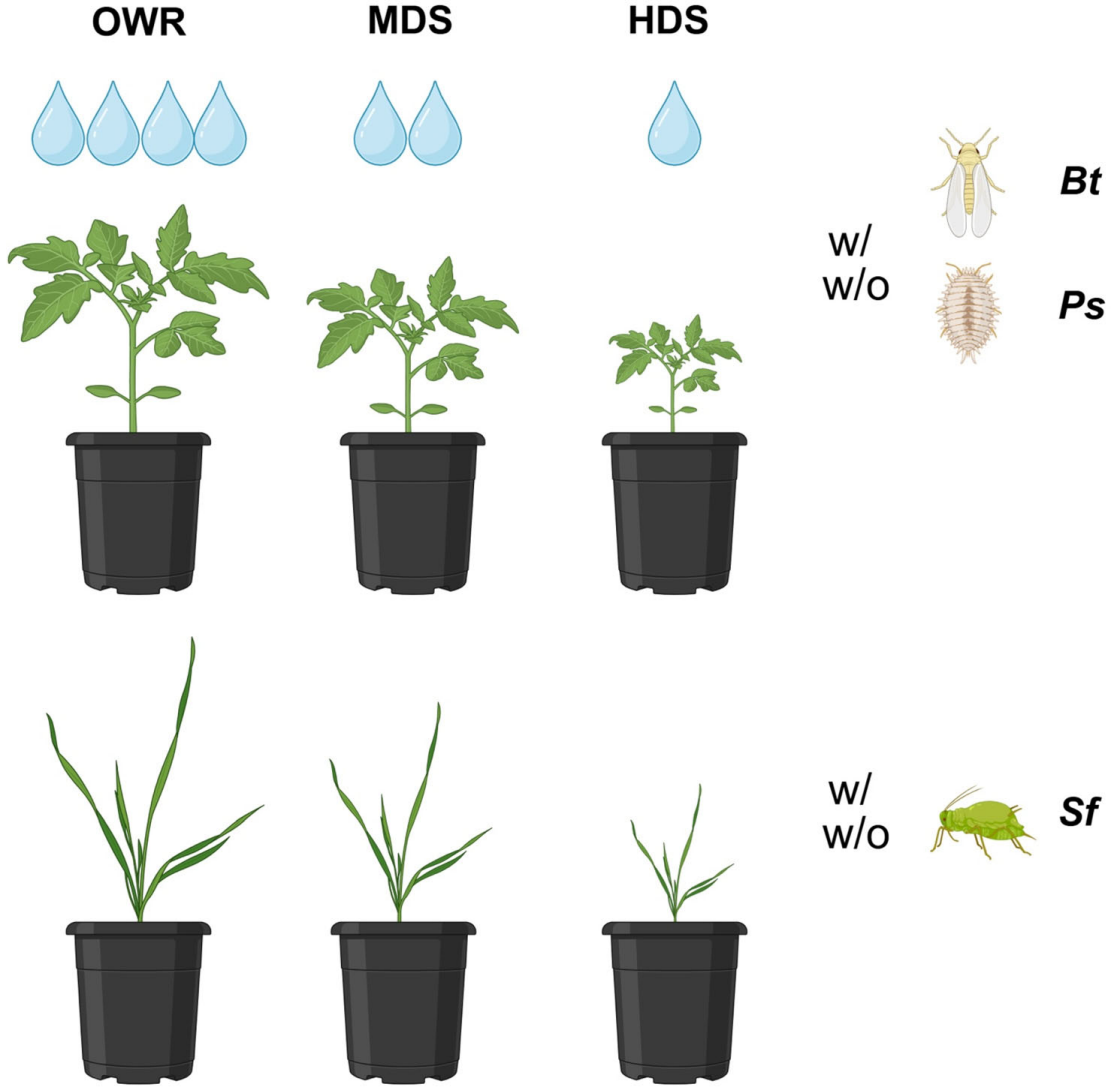
Experimental set‐up of tomato and wheat plants under different water regimes (OWR, optimal water regime; MDS, medium drought stress; HDS, high drought stress) with or without pest infestation (*Bt*, *Bemisia tabaci; Ps, Phenacoccus solenopsis*; *Sf*, *Sitobion fragariae*) tested in dual choice and gene expression assays.

Tomato and wheat plants grown under different water regimes were arranged into six different experimental groups (Fig. 1), combining various plant‐insect conditions. Specifically, for tomato plants, both combinations related to infestation with *B. tabaci* and *P. solenopsis* were considered with the following treatments: uninfested OWR plants; uninfested MDS plants; uninfested HDS plants; OWR + *P. solenopsis* or *B. tabaci*‐infested plants; MDS + *P. solenopsis* or *B. tabaci*‐infested plants and HDS + *P. solenopsis* or *B. tabaci*‐infested plants. The following tested treatments were considered with wheat plants: uninfested OWR plants; uninfested MDS plants; uninfested HDS plants; OWR + *S. fragariae*‐infested plants; MDS + *S. fragariae*‐infested plants and HDS + *S. fragariae*‐infested plants.

#### 2.2.1 Phenotypic plant response

In order to evaluate plant morphological response to water stress, 15 tomato and 15 wheat plants were selected 30 days after planting for each water regime. The following morphological parameters were measured: height from collar, stem diameter, root length, FW and DW. Collar height and stem diameter were measured with a ruler and vernier caliper, respectively. Three weightings were carried out for each sample of leaves, stem and roots. After separating the different plant organs with scissors, their FW was measured on a 10‐mg‐sensitive microbalance (Mettler‐Toledo GmbH, Giessen, Germany). Plant DW was measured after the introduction of separated plant organs into filter paper bags in a desiccator set at 60 °C for 72 h.

#### 2.2.2 Gene expression

The transcriptional response of major defense genes involved in abiotic and biotic stress in tomato and wheat was analyzed by using the following marker genes: the abscisic acid stress ripening protein (*ASR1*) that encodes a key protein involved in abscisic acid (ABA) signaling pathway, the pathogenesis‐related gene (*PR1*) that regulates the salicylic acid (SA) pathway, and the proteinase inhibitors 2 (*PIN2*) and lipoxygenase (*LOX*) which are involved in jasmonic acid (JA) signaling pathways conferring insect resistance in tomato and wheat, respectively. To minimize wounding‐ and dehydration‐induced gene expression, apical leaf samples from tomato and wheat were cut with a sterile blade and immediately frozen in liquid nitrogen (N_2_). Frozen samples were ground to a fine powder in a mortar with a ceramic pestle while submerged in liquid N_2_. Total RNA was isolated from 30 mg of each leaf sample using the SV Total RNA™ Isolation System (Z3100; Promega Corp., Madison, WI, USA) according to the manufacturer's instructions. RNA concentration and purity were determined with a NanoDrop™ Lite Spectrophotometer (ND‐LITE‐PR; Thermo Fisher Scientific, Waltham, MA, USA). First‐strand cDNA was synthesized from 500 ng RNA using a Reverse Transcription System kit (A1250; Promega Corp.). The transcript levels of *ASR1*, *PIN2*, *LOX* and *PR1* were quantified with quantitative real‐time (qRT)‐PCR. Elongation Factor (*EF1*) and Actin (*ACT*) were used as reference genes to normalize qRT‐PCR amplification for *S. lycopersicum* and *T. aestivum*, respectively. Primers used in qRT‐PCR amplification are listed in Table 1. The amplification reactions were performed in a final volume of 10 μL containing 5 μL SensiFAST™ SYBR® & Fluorescein mix (2×), 0.4 μL of each gene‐specific primer (10 mm) and 1 μL cDNA first‐strand template. Thermal cycling conditions were: 95 °C for 120 s; 40 cycles of 95 °C for 5 s; 60 °C for 10 s; and 72 °C for 5 s. The fold changes of the target genes were calculated using the 2^−ΔΔCt^ method. The reactions were carried out in AriaMx Real‐Time PCR System (Agilent Technologies, Santa Clara, CA, USA). Each treatment combination (i.e. each water regime with or without herbivore infestation) was performed with three biological replicates (i.e. three plants). Each biological replicate was analyzed in duplicate (i.e. two technical replicates) in the qRT‐PCR reactions.

**Table 1:** qRT‐PCR primer sequences of genes involved in abscisic acid (ABA), jasmonic acid (JA) and salicylic acid (SA) defense responses for tomato and wheat

Plant	Gene	Function	Primer sequence (5′‐3′)
**Tomato**	***EF1*‐F**	*Housekeeping*	CCTGGACAGATTGGAAATGG
	***EF1*‐R**		GACCACCTGTCGATCTTGGT
	***ASR1*‐F**	*ABA signaling pathway*	TGTGCAATTTGTCTTGTGGAA
	***ASR1*‐R**		CGGACATGACGAGTTCGATA
	***PIN2*‐F**	*JA signaling pathway*	CTTGCCCCAAGAATTGTGAT
	***PIN2*‐R**		GCCCTAGCGTATTACGGAGA
**Wheat**	***ACT*‐F**	*Housekeeping*	GGAAAATCAGTCTCGGTTCAG
	***ACT*‐R**		TCATACAGCAGGCAAGCAC
	***LOX*‐F**	*JA signaling pathway*	GACCAGCGAAACAACAACC
	***LOX*‐R**		GCATACAATAGCGGGAACAC
	***PR1*‐F**	*SA signaling pathway*	ATAACCTCGGCGTCTTCAT
	***PR1*‐R**		TACTCGCTCGGTCCCTCT

#### 2.2.3 Insect olfactory responses

The behavioral responses of *E. formosa*, *C. montrouzieri* and *A. colemani* females to plant‐emitted volatiles were investigated in a two‐way olfactometer (VidraFOC, Barcelona, Spain). The olfactometer consisted of a Y‐shaped glass tube. For *C. montrouzieri*, the Y‐tube had an internal diameter of 3.5 cm with an entry arm 13 cm long and two side arms 13 cm long, forming a 75° inside angle. For *E. formosa* and *A. colemani*, the Y‐tube had an internal diameter of 1 cm with an entry arm 8.5 cm long and two side arms 7.5 cm long, forming a 60° inside angle. The base of the Y‐tube was connected to an air pump that provided a unidirectional carbon‐filtered airflow at 150 ± 1 mL min^−1^. The arms were connected via semi‐transparent PVC tubes to two identical glass jars (5 L volume) containing a test odor source. Four 60‐cm‐long fluorescent tubes (L18 W/765; OSRAM GmbH, Berlin, Germany) were positioned 40 cm above the arms. For each insect species, a single female was introduced into the entry arm of the tube and observed until it made a choice, defined as walking at least halfway into one of the side arms. For each odor source combination, a total of 56 females were tested (14 females/plant combination replicated four times). After testing seven females, the olfactometer arms were flipped by 180° to minimize the spatial bias. After testing 14 females, the plants were changed and the olfactometer setup was rinsed with neutral soap, water and acetone among the tested treatments. The maximum time allowed for insects to make a choice in the olfactometer was 10 min for *E. formosa* and 5 min for *C. montrouzieri* and *A. colemani*. Females that did not make a choice within the maximum time allowed were recorded as ‘no‐choice’ and were excluded from the dataset for analyses. Each individual was tested only once. The environmental conditions in the Y‐tube experiments were 23 ± 2 °C and 60 ± 10% RH.

### 2.3 Data analysis

The Kolmogorov–Smirnov test and the Levene's test were used to assess the raw datasets for normality and homogeneity of variance, respectively. The effect of the three different water regimes (OWR, MDS, HDS) on plant morphological parameters (height from collar, stem diameter, FW and DW) was analyzed using one‐way ANOVA followed by the least significant difference (LSD) *post hoc* test for mean comparisons (*P* < 0.05). For qRT‐PCR, the fold‐changes in the expression of target genes (*ASR1*, *PIN2*, *LOX*, *PR1*) were calculated using the 2^−△△Ct^ normalization method.^25^ The effect of tested factors (water regime and pest presence) and their interaction on the transcriptional responses of *ASR1*, *PIN2*, *LOX* and *PR1* genes were analyzed through a multifactorial ANOVA followed by LSD *post hoc* test (*P* < 0.05) to highlight significant mean differences among the treatments. Insect olfactory choices were analyzed using chi‐square (χ^2^) test to determine if the distribution of side‐arm choices between pairs of odors significantly deviated from the null expectation of equal frequency (1:1) for odor source selection. Statistical analyses were performed using IBM spss statistics v23 (released 2015; IBM Corp., Armonk, NY, USA).

## 3 RESULTS

### 3.1 Effects of water stress on plants

**Figure 2:**
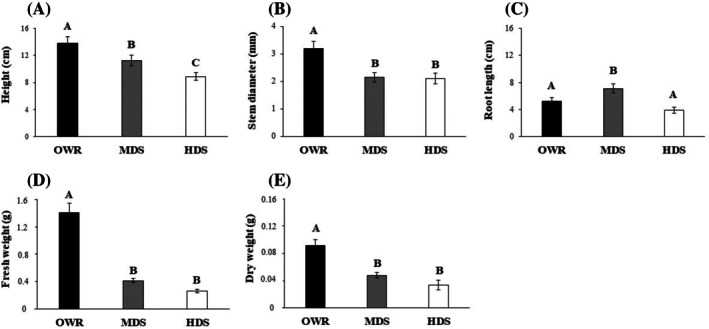
Effect of different water regimes on tomato morphology: height from collar (A), stem diameter (B), root length (C), FW (D) and DW (E). Different letters over the bars indicate significant differences at *P* < 0.05 (ANOVA; LSD test).

**Figure 3:**
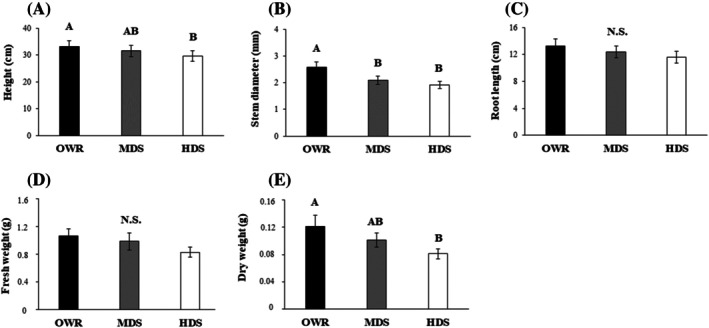
Effect of different water regimes on wheat morphology: height from collar (A), stem diameter (B), root length (C), FW (D) and DW (E). Different letters over the bars indicate significant differences at *P* < 0.05 (ANOVA; LSD test).

In tomato plants, height from collar showed significant changes among plants irrigated with different water regimes (*F*_2,44_ = 38.104; *P* < 0.001). Also the stem diameter was significantly reduced under MDS and HDS water regimes (*F*_2,44_ = 8.114; *P* < 0.001). With regard to root length, plants under MDS showed a significantly greater length compared to those under OWR and HDS, (*F*_2,44_ = 12.225, *P* < 0.001). A significant difference was recorded between drought‐stressed and control tomato plants for FW (mean values varied from 0.27 ± 0.03 g for HDS to 1.40 ± 0.15 g for OWR) (*F*_2,44_ = 10.046, *P* < 0.001). A similar trend was observed for the DW with mean values ranging from 0.03 ± 0.01 g for tomato plants under HDS and 0.09 ± 0.01 g for OWR plants (*F*_2,44_ = 61.243; *P* < 0.001) (Fig. 2). In wheat, the height from the collar showed significant differences between HDS and OWR plants (*F*_2,44_ = 5.939; *P* < 0.001). The stem diameter significantly varied between drought‐stressed (HDS and MDS) and OWR plants (*F*_2,44_ = 17.531; *P* < 0.001). Conversely, neither length root (*F*_2,__44_ = 2.507; *P* = 0.094), nor plant FW (*F*_2,44_ = 2.025; *P* = 0.144) had significant variation under the three tested water regimes. However, the DW significantly decreased under HDS in comparison to OWR plants (*F*_2,44_ = 3.527; *P* = 0.038) (Fig. 3).

### 3.2 Gene expression analysis

**Figure 4:**
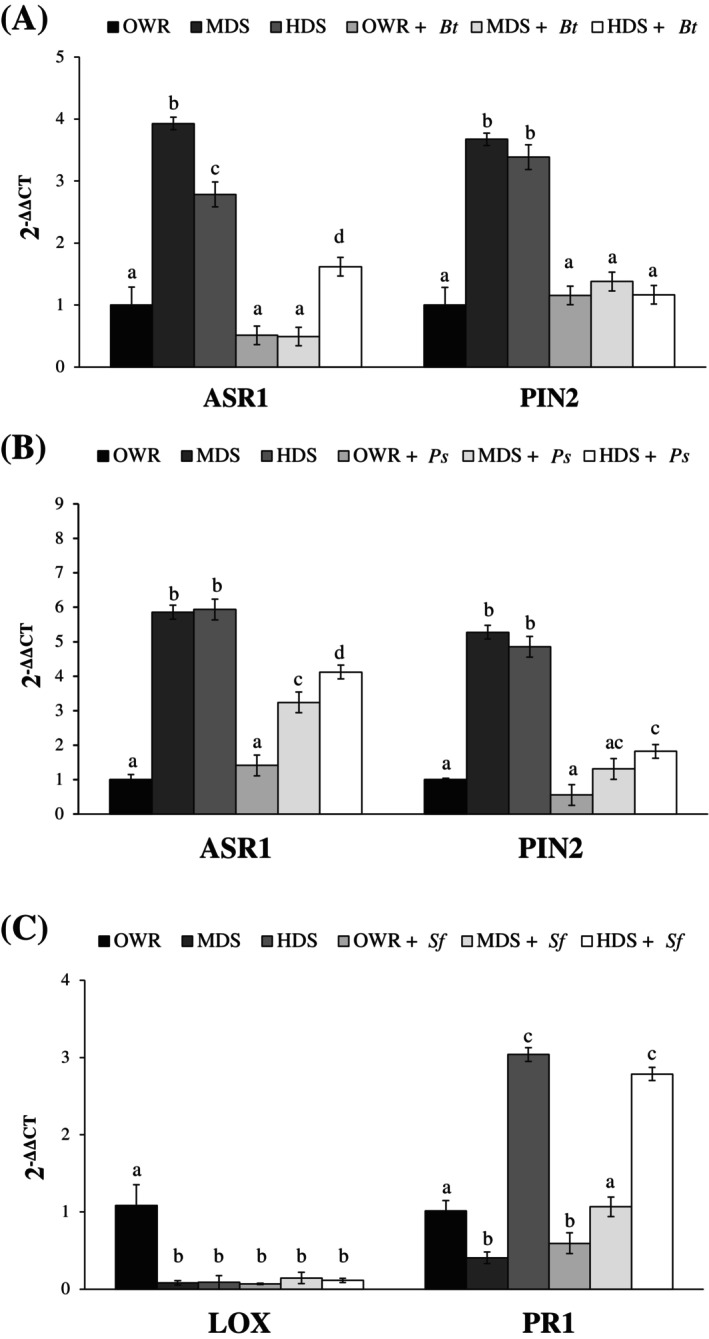
Expression levels of *ASR1* and *PIN2* genes in tomato which are (respectively) abscisic acid (ABA)‐ and jasmonic acid (JA)‐responsive, with different water regime conditions and/or *Bemisia tabaci* (*Bt*) (A) or *Phenacoccus solenopsis* (*Ps*) (B) infestation; expression levels of *LOX* and *PR1* target genes in wheat (C), which are respectively jasmonic acid (JA)‐ and salicylic acid (SA)‐responsive, with different water regime conditions and/or *Sitobion fragariae* (*Sf*) infestation. To determine the relative expression level of target genes, the ΔΔCt method was used,^25^ in which values are normalized from housekeeping gene *EF1*. Target gene is considered overexpressed when 2^−ΔΔCT^ ≥ 2. Data are presented as mean (± SE) of three independent analyses of transcript expression relative to housekeeping gene plants (*n* = 3). Bars for treatment bearing the same letters did not differ at *P* < 0.05 (ANOVA, LSD test).

In tomato plants, the expression level of *ASR1* was significantly affected by the water regime (*F*_2,18_ = 14.279; *P* < 0.001), the presence of *B. tabaci* (*F*_1,18_ = 44.057; *P* < 0.001) and their interaction (*F*_1,18_ = 12.217; *P* < 0.001). In the experiment with noninfested plants by *B. tabaci*, *ASR1* gene was significantly overexpressed by ≈ 4‐fold and 2.5‐fold for tomato plants grown under MDS and HDS conditions, respectively. The high drought‐stress and infested plants (HDS + *Bt*) also showed overregulation of the *ASR1* gene [Fig. 4(A)]. The *PIN2* gene (marker for JA) was 3.5‐fold upregulated in tomato plants grown under MDS and HDS conditions in comparison to the control (OWR) (*F*_2,18_ = 29.493; *P* < 0.001). However, the presence of *B. tabaci* caused no overregulation of JA when the infestation was associated with different drought stress.

In water‐stressed tomato plants infested or not with *P. solenopsis* [Fig. 4(B)], MDS and HDS conditions induced a significant upregulation of *ASR1* gene (*F*_5,18_ = 5.2, *P* = 0.009), whereas a ≈ 5–6‐fold increase in *PIN2* expression was recorded only under water‐stress regimes (*F*_5,18_ = 3.672; *P* = 0.03).

In *T. aestivum* [Fig. 4(C)], a statistically significant effect of the water regime (*F*_2,18_ = 5.967; *P* = 0.016) was recorded on *PR1* gene expression. Namely, *PR1* levels increased by ≤3‐fold when plants were grown under HDS with or without *S. fragariae* infestation, however neither the pest nor its interaction with water condition affected the transcriptomic level. Similar water stress conditions caused a significant underexpression of the *LOX* gene (*F*_2,18_ = 10.725; *P* < 0.002) and this effect was exacerbated by the presence of the pest (*F*_1,18_ = 10.342; *P* = 0.007) and its interaction with water deficit condition (*F*_2,18_ = 13.466; *P* < 0.001).

### 3.3 Insect behavioral responses

In a two‐way olfactometer, although no statistical differences were highlighted, *E. formosa* females equally preferred plant cues emitted by *B. tabaci*‐infested tomatoes under MDS compared to infested plants under OWR and *B. tabaci*‐infested plants under OWR were more attractive in comparison to those grown under HDS.

**Figure 5:**
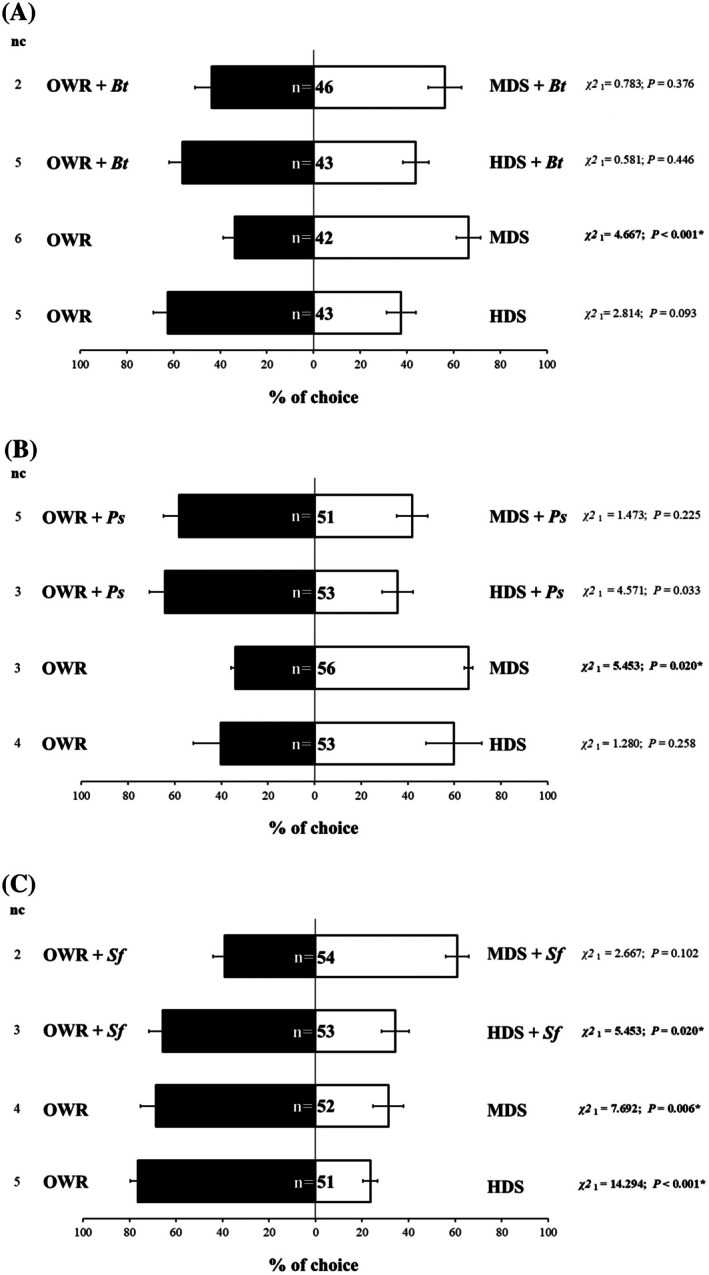
Behavioral response of *Encarsia formosa* (A), *Cryptolaemus montrouzieri* (B) and *Aphidius colemani* (C) females in dual choice test when exposed to different conditions of water regime and/or insect infestation. n, total number of respondent females; nc, number of individuals that made no choice; OWR, optimal water regime; MDS, medium drought stress; HDS, high drought stress; *Bt*, *Bemisia tabaci*; *Ps*, *Phenacoccus solenopsis*; *Sf*, *Sitobion fragariae*. Significant differences based on a chi‐square test are marked with (*) (*P* < 0.05).

When comparing plants subjected to different water regimes when no pest infestation occurred, *E. formosa* preferred plants under OWR conditions when compared to HDS, whereas no significant differences were observed. Conversely, in the comparison between MDS and OWR plants, the tested *E. formosa* females significantly preferred plant cues emitted by tomatoes grown under MDS conditions (χ^2^_1_ = 4.667, *P* < 0.001) [Fig. 5(A)]. In dual choice olfactometer assays with noninfested plants, *C. montrouzieri* females showed a significant preference for volatiles emitted by plants under MDS (χ^2^_1_ = 5.453; *P* = 0.020), and a similar trend was observed for HDS plants. However, when comparing *P. solenopsis* infested tomato plants, *C. montrouzieri* significantly preferred OWR plants over HDS plants (χ^2^_1_ = 5.453; *P* = 0.033). Likewise, although no statistically significant differences were observed, the predators preferred OWR‐infested plants to plants grown under MDS conditions [Fig. 5(B)]. *A. colemani* females showed a statistically significant preference for the volatiles emitted by wheat plants under OWR with or without *S. fragariae* infestation in all comparisons, except when OWR was tested against MDS in the presence of *S. fragariae* [Fig. 5(C)].

## 4 DISCUSSION

Understanding how natural enemies respond to abiotic stressors such as drought, alone or in combination with herbivore pests, is crucial for improving pest management strategies within IPM programs.^26^ In this study, we tested the hypothesis that altered water inputs and herbivore infestation could trigger bottom‐up effects leading to plant morphological and transcriptomic changes, ultimately affecting the olfactory responses of natural enemies. Overall, our results partially support this hypothesis. Water regimes significantly affected plant morphological traits and modulated the expression of defense‐related genes in both tomato and wheat. Additionally, the combination of water stress and herbivore presence altered the olfactory responses of the natural enemies *E. formosa*, *C. montrouzieri* and *A. colemani* to plant‐emitted volatiles. These findings indicate that drought conditions can influence multitrophic interactions by modifying plant traits and volatile‐mediated signaling, thereby potentially affecting the efficiency of biological control agents.

Drought stress poses a serious threat to agriculture, severely restricting plant development.^8^ In our investigation, tomato plants grown under drought stress exhibited significant reductions in stem plant height, diameter, DW and root length compared to plants grown under optimal water conditions. Supporting these findings, Dong *et al*.^9^ reported how water stress can reduce tomato growth, particularly affecting plant height. Likewise, Han *et al*.^10^, ^18^ provided further evidence supporting the detrimental impacts of drought on tomato growth, reporting a reduction in plant height and a compensatory elongation of roots under drought conditions. Real‐Santillán *et al*.^27^ demonstrated that plants receiving higher levels of water irrigation showed longer shoots and higher DW. In agreement with these findings, our results revealed a significantly higher DW in tomato plants under OWR conditions, despite FW not being significantly affected. The differences among these studies are likely to be related to the number of observation days, the specific watering regimes applied and the peculiar environmental conditions of the respective experiments.

In our experimental trials, wheat plants under high drought stress showed significant reductions in collar height and DW compared to the control group. These findings are consistent with Abid *et al*.,^28^ who showed a negative relationship between drought intensity and plant growth together with dry matter in wheat plants. Al‐Maskri *et al*.^29^ and Alsamadany *et al*.^30^ also demonstrated that drought stress negatively affects the growth and development of wheat plants. Likewise, Alexieva *et al*.^31^ provided evidence that water limitation leads to a decrease in the DW of wheat plants. Taken together, these results highlight the overall vulnerability of both tomato and wheat plants to water scarcity and the significant impact of drought on plant biomass productivity,^32^ impeding the growth and development of both species.^33^, ^34^

Under drought stress, plant defense signaling is characterized by extensive transcriptional metabolic changes.^8^, ^35^ In our study, a medium to high water deficit in tomato plants is likely to have activated the ABA and JA signaling pathways, as a result of the significant upregulation of the *ASR1* and *PIN2* genes. As reported previously, water stress induces changes in the metabolites of tomato plants,^11^, ^36^ leading to the regulation of gene expression through transcription factors that are stimulated by drought conditions.^8^ According to Cutler *et al*.^37^ and Arbona *et al*.,^13^ drought‐stressed plants released an increased level of ABA biosynthesis to initiate signal transduction, enabling plants to prevent water loss and maintain a sufficient water level during stress conditions.

In wheat plants developed under high drought stress conditions, a significant activation of the *PR1* gene associated with the SA signaling pathway was observed. ABA has been observed to be the primary stress signal pathway involved in the physiological response in wheat under drought stress through the expression of drought‐responsive genes to maintain plant functions and stability.^38^ Under similar circumstances, SA accumulation plays a crucial role in regulating plant system function and mitigating cell water loss.^39^ This divergence in gene expression between the two plants highlights the species‐specific adaptations to stress.^40^ Plants also can modulate their responses when faced with both biotic and abiotic stress, resulting in a more complex and diverse set of responses compared to single stresses.^41^ In our research, the presence of *B. tabaci* or *P. solenopsis* differently influenced the activation of ABA and JA pathways in tomato plants. A significant upregulation of *ASR1* in highly drought‐stressed tomatoes infested with *B. tabaci* (HDS + Bt) and in highly and moderately water‐stressed plants infested with *P. solenopsis* (MDS or HDS + Ps) was observed. Likewise, Arbona *et al*.,^13^ found tomato plants under moderate drought stress alone and combined with *Tetranychus evansi* Baker and Pritchar (Tetranychidae) infestation showed higher levels of SA that regulate plant responses when abiotic and biotic stresses are combined. These findings suggest that when different types of stress interact, there is a crosstalk between their signaling pathways, which can exhibit synergistic or antagonistic responses in plant defenses, highlighting the intricate regulatory mechanisms that plants use to survive harsh environmental conditions.^42^

Wheat plants also have evolved physiological and biochemical modifications as a strategy to tackle water scarcity.^43^ These adaptations involve adjustments in plant metabolism^44^ that are achieved through the activation of multiple signaling pathways for the regulation of gene expression and maintenance of cellular water balance.^45^, ^46^ Our investigation showed that wheat plants facing the combination of a high water deficit and the presence of *S. fragariae* resulted in the overexpression of the *PR1* gene, which is involved in the SA pathway, compared to control conditions. However, neither abiotic nor biotic stress over‐regulated the *LOX* gene, which is involved in the activation of the JA pathway. This result was rather expected, as several pieces of evidence suggest an antagonistic crosstalk between the SA and JA pathways.^47^ Likewise, Caarls *et al*.,^48^ observed that the SA signaling pathway affects JA‐induced transcription by causing the degradation of transcription factors, which directly or indirectly inhibits the expression of JA‐responsive genes.

Plants release volatile cues in reaction to drought and biotic stress, aiding in environmental adaptation and functioning as infochemicals in multitrophic networks.^49^, ^50^ These compounds can influence biocontrol efficacy owing to cascading effects from plant–herbivore–natural enemy interactions, shaping the behavior of beneficial organisms.^27^ In our experiment, the parasitoid *E. formosa* showed a significant preference for plants under MDS conditions compared to the control, regardless of *B. tabaci* infestation. Despite not being statistically confirmed, *E. formosa* seemed to prefer optimally irrigated plants over highly drought‐stressed plants, independently of *B. tabaci* infestation. This suggests that the water regime, especially MDS, exerts a dominant effect on parasitoid choice. The results also are consistent with the significant over‐regulation of *ASR1* and *PIN2* genes under MDS. The increased expression of ABA‐ and JA‐related marker genes under MDS conditions suggests that water limitation modulates plant physiological responses in ways that affect volatile emissions. These transcriptional changes are consistent with the altered volatile profiles released by stressed plants and may help explain the increased attraction of natural enemies observed in olfactometer assays. These behavioral observations are aligned with previous studies documenting that water stress can elicit an array of plant modifications, including the plant's capacity to synthesize and release volatiles.^51^ These compounds can change in response to combined biotic and abiotic stressors, leading to increased emission of volatile molecules that attract insect natural enemies.^52^, ^53^ Yet, insect‐derived elicitors, often released through oviposition and salivary compounds, can induce distinct volatile plant profiles which help parasitoids locate their hosts.^54^ Moreover, the co‐occurrence of stressors in tomato plants, such as drought stress and pest infestation, can lead to an increase in the biosynthesis and emission of volatile compounds.^20^ These molecules serve as potent defensive compounds and mediate plant–plant communication with their surrounding environment, including beneficial organisms. Variations in plant metabolites from parasitized or nonparasitized herbivore‐infested plants can have notable impacts on the effectiveness of parasitoid activity.^2^ In pest‐free tomato plants *C. montrouzieri* females exhibited a significant preference for plants grown under MDS compared to those grown under OWR, and a similar result was observed under HDS conditions although no statistical evidence occurred. By contrast, female predators significantly preferred optimal water regimes in the presence of *P. solenopsis* infestation. Once again, water stress regimes dominated predator host selection, as evidenced by a significant gene over‐regulation of tomato defense pathways in both MDS and HDS conditions. These results support the hypothesis that plants respond to water limitation by increasing the emission of volatile compounds as an adaptive mechanism to cope with their environment and prolong their life cycle.^55^ Emitted volatile compounds can play a crucial role in enhancing the biological control activity of natural enemies by aiding them in identifying specific plant species upon disturbance by phytophagous insects.^56^ The emission of herbivore‐induced plant volatiles occurs in response to herbivore salivary secretions, oviposition and excrement, thus attracting predators and allowing them to find their hosts on a large scale.^54^, ^56^ Han *et al*.^18^ discovered that drought harmed the predation efficacy of *Macrolophus pygmaeus* (Rambur) (Hemiptera: Miridae), a key predatory insect in tomato cropping systems. The study revealed a 30% reduction in the longevity of the predator under drought conditions. The extent of this impact was found to be influenced by the growth status of the plants.

In our study, *C. montrouzieri* females preferred volatiles from well‐watered tomato plants infested with *P. solenopsis* compared to those from water‐stressed‐infested plants. These findings suggest that plants respond to herbivores by releasing volatile substances that attract predatory arthropods.^57^

Many volatile molecules from the wheat plant, *T. aestivum*, have been identified to be involved in tri‐trophic interactions and affect the behavior of beneficial insects.^58^ In our experiment, the parasitoid *A. colemani* showed a significant preference for wheat plants under optimal water conditions, regardless of *S. fragariae* infestation, in all tested combinations except for the MDS infested plants. These results suggest that, in a similar way to *E. formosa*, parasitoids are most influenced by the presence of their target pest on the host plant, in contrast to the predator *C. montrouzieri* where water stress was the only determining factor. Xie *et al*.^58^ demonstrated that the emitted volatiles from wheat plants attract the predators *Episyrphus balteatus* (De Geer) (Diptera: Syrphidae) and *Harmonia axyridis* (Pallas) (Coleoptera: Coccinellidae). Gouinguene & Turlings^59^ provided evidence that drought stress increased volatile emissions in *Zea mays* L. plants, whereas drought‐stressed infested plants emitted more volatile compounds that attract natural enemies. Likewise, Salerno *et al*.,^60^ in a bioassay model using *Vicia faba* L. plants under water stress alone and in combination with herbivore infestation, demonstrated that the simultaneous occurrence of biotic and abiotic stresses can lead to changes in volatile profiles emitted by plants. These altered volatile profiles were found to affect the behavior of the egg parasitoid of *Nezara viridula* L. (Hemiptera: Pentatomidae), enhancing its attraction toward stressed‐infested plants. This evidence supports the hypothesis that the combination of biotic and abiotic stresses may have a synergistic effect on the emission of volatile compounds, which in turn influences the interactions between plants and their associated natural enemies.^20^, ^61^ The different responses of natural enemies to drought stress in wheat and tomato plants could contribute to the less clear effect of pest presence on natural enemy selection in wheat than in tomatoes. In wheat, the preference of *A. colemani* for well‐watered conditions may exclude the direct effect of pest presence on natural enemy selection. The different volatile profiles emitted by wheat plants under drought stress and pest infestation could potentially interfere with the ability of natural enemies to distinguish between pest‐infested and uninfested plants by altering the plant signals normally induced by pests. This complexity in volatile emissions and natural enemy responses in wheat may introduce additional variables that make the effect of pest presence on natural enemy selection less straightforward compared to the more evident responses observed in tomato plants. Moreover, although in our study we observed an over‐regulation of the *PR1* gene under HDS conditions, the parasitoid still preferred well‐watered plants, an aspect that needs to be explored further in the future. The responses of natural enemies to drought stress highlight the species‐specific nature of their interactions with plants. *E. formosa*'s preference for MDS‐exposed tomato plants suggests an attraction to the unique volatile compounds emitted by stressed tomato foliage. By contrast, *A. colemani*'s preference for well‐watered wheat plants indicates a specific adaptation to optimal watering conditions within the wheat crop. These distinct preferences underscore how natural enemies may have evolved to respond differently to stressors based on the plant species involved, owing to variations in volatile profiles and plant signaling mechanisms. The changes in plant volatile emissions induced by stressors such as drought and herbivore infestations are crucial in mediating these interactions, as they serve as chemical signals that guide natural enemies toward their host or prey.^62^ By influencing the behavior of beneficial organisms, these volatile compounds play a vital role in shaping the dynamics of agroecosystems and enhancing biological control strategies in agricultural systems.^63^

## 5 CONCLUSIONS

Overall, our findings highlight the importance of investigating the combined effects of various stressors on plant defenses and the resulting behavioral changes in multitrophic systems owing to bottom‐up effects. Although these insights can contribute to developing plant protection strategies within IPM approaches for agricultural crops,^11^ further research is needed. For instance, analyzing volatile compounds emitted by plants under different stress combinations and quantifying plant defenses^64^ can help in understanding the molecular mechanisms involved in multitrophic interactions in order to determine the efficacy of biocontrol programs. Moreover, field trials aiming to validate the integration of stress‐induced plant defenses in pest management strategies, considering the effect of climate change on plant defenses, could provide a more comprehensive and predictive understanding of agroecosystems.^65^ From an applicative point of view, our results suggest that moderate water stress does not necessarily compromise, and may even enhance the effectiveness of biological control agents by strengthening the indirect defenses of plants. Integrating irrigation management with biological control programs could therefore represent a valid strategy for improving pest management under the water deficit conditions expected in future climate scenarios. Such an approach may help in enabling technicians to make rational decisions, promote ecosystem resilience and preserve its services.^66^ In conclusion, a multidisciplinary approach undoubtedly provides valuable knowledge allowing for more accurate predictions on future biocontrol services and the development of sustainable and more targeted IPM strategies.

## CONFLICT OF INTEREST

The authors declare that they have no conflict of interest.

## AUTHOR CONTRIBUTIONS

Mariangela Milordo: Investigation, Methodology, Formal analysis, Visualization, Writing–original draft. Eya Ben Hmad: Investigation, Writing–review and editing. Maria Flavia Pitruzzello: Investigation, Writing–review and editing. Carmelo Cavallaro: Investigation, Writing–review and editing. Giuseppe Massimino Cocuzza: Funding acquisition, Writing–review and editing. Lucia Zappalà: Funding acquisition, Writing–review and editing. Peng Han: Conceptualization, Writing–review & editing. Antonio Biondi: Conceptualization, Funding acquisition, Supervision, Writing–review and editing. Michele Ricupero: Conceptualization, Funding acquisition, Supervision, Writing–review and editing.

## Data Availability

The data that support the findings of this study are available from the corresponding author upon reasonable request.
